# Autologous micro-fragmented adipose tissue associated with arthroscopy in moderate–severe knee osteoarthritis: outcome at two year follow-up

**DOI:** 10.1186/s12891-022-05921-6

**Published:** 2022-11-08

**Authors:** Andrea Giorgini, Filippo Selleri, Francesco Zambianchi, Giacomo Cataldo, Elena Francioni, Fabio Catani

**Affiliations:** Department of Orthopaedic Surgery, Azienda Ospedaliero Universitaria di Modena, University of Modena and Reggio-Emilia, Modena, Italy

**Keywords:** Knee, Osteoarthritis, micro-fragmented adipose tissue, Intra-articular injection, Arthroscopy, Cartilage

## Abstract

**Background:**

Adipose tissue has recently gained growing interest in the treatment of osteoarthritis (OA). The aim of the present study was to evaluate the efficacy of a single injection of autologous micro-fragmented adipose tissue (aMFAT) associated with arthroscopy (cartilage debridement/meniscal regularization or selective meniscectomy/micro-drilling) for symptomatic knee OA.

**Methods:**

This retrospective, single-center study included 49 patients (50 knees) affected by knee OA (radiographic Kellgren-Lawrence III-IV) treated with a single injection of autologous micro-fragmented adipose tissue and knee arthroscopy. Knee Injury and Osteoarthritis Outcome Score (KOOS) and subjective International Knee Documentation Committee (IKDC) score were the primary outcome measures and were collected at one and 2 years post-operatively. Patients were divided into clusters based on age, complexity of arthroscopic procedures and chondral lesion grade.

**Results:**

Four patients underwent knee replacement (8%). No major adverse events were reported. Minimal Clinically Important Difference (MCID) for KOOS and IKDC was reached by 84 and 74% of all cases at 1 year and by 80 and 76% at 2 years, respectively. High grade chondral lesions negatively affected the outcome at 2 years follow-up (*p* < 0.05 for IKDC, KOOS overall and 3 out of 5 subscales).

**Conclusion:**

The injection of micro-fragmented adipose tissue associated with arthroscopy demonstrated to be a safe and effective procedure for the treatment of knee OA, with a substantial improvement in IKDC and KOOS scores and without major complications.

**Supplementary Information:**

The online version contains supplementary material available at 10.1186/s12891-022-05921-6.

## Background

Knee osteoarthritis (OA) is a common disease causing pain and reducing quality of life in a high number of patients [1]. Non-operative treatments have shown relative and short-lasting effect on pain relief [2]. Intra-articular injection of corticosteroids [3], blood-derived products such as platelet rich plasma (PRP) [4, 5], autologous protein solution (APS) [6] and mesenchymal stem cells (MSCs) [7, 8] have all shown promising results for the treatment of knee osteoarthritis at early stages. However, partial or total knee arthroplasty represents the gold standard treatment for end-stage knee OA [9, 10].

More recently, adipose tissue has gained growing interest as a source of MSCs. In fact, adipose derived stem cells are 5% of nucleated cells versus 0.0001–0.01% of bone marrow-derived stem cells [11]. Mechanical and enzymatic methods have been proposed for adipose MSCs processing and transfer. Regulatory issues within the European Community greatly limit the use of enzymatic procedures which could deliver a product with higher cell viability and differentiative potential compared to non-enzymatic methods [12]. However, various authors [13, 14] advocate the important role played by extracellular matrix, which is preserved via mechanical processing. Recently introduced systems offer easy harvesting (according to Coleman technique [15]), processing and transfer of refined autologous micro-fragmented adipose tissue (aMFAT), without expansion and/or enzymatic treatment [16]. Different mechanical methods might offer a final product with some difference in terms of cell viability and differentiative potential [12, 17].

Even if the use of aMFAT obtained via mechanical method in the treatment of knee OA has shown promising results [18–21], data relative to clinical outcome and efficacy are lacking.

Therefore, the main purpose of the present single-center, retrospective, observational study was to determine the clinical outcomes of patients who received a single injection of aMFAT associated with arthroscopy for symptomatic Kellgren Lawrence (KL) III and IV knee OA. The secondary aim of the present study was to identify which patient benefits the most out of this procedure. The efficacy and safety of the procedure were also assessed.

## Methods

### Study design

This retrospective, observational, single center study included a series of 50 consecutive knees treated with a single injection of aMFAT (Lipogems® Ortho Kit, Lipogems International SpA, Milan, Italy) and knee arthroscopy for symptomatic knee OA between December 2015 and February 2018. All patients were previously given the indication for prosthetic knee replacement but refused the procedure. Two surgeons with extensive experience in arthroscopic procedures performed all the operations. Patients aged 18 to 80 were screened and considered eligible for study inclusion if they had received a diagnosis of severe knee pain and radiographic documentation of primary, post-traumatic or post-meniscectomy Kellgren Lawrence III and IV knee OA. The exclusion criteria were coronal limb deformity with anatomical femoro-tibial angle (aFTA) < 181° and > 191°, as measured on short-view preoperative weight bearing knee radiographs, cartilage defects > 4 cm^2^ as documented on preoperative MRI or intra-operatively, vasculitis and other vascular diseases, neuromuscular disorders, active or previous knee infection, previous open knee fractures, recent trauma and intra-articular injection less than 6 months before the treatment.

### Surgical procedure

Adipose tissue was harvested from the abdomen in all patients, except in one case in which it was harvested from the thigh, due to the paucity of abdominal adipose tissue. The harvesting procedure was performed by a plastic surgeon or by a trainee orthopedic surgeon. After harvesting, the adipose tissue was processed according to the manufacturer’s procedure as previously reported [14, 22]. In the meantime, arthroscopy was performed. All knees underwent joint lavage and debridement at first, then, if present, meniscal lesions or isolated, femoral, weightbearing, Outerbridge grade IV chondral defect < 4 cm^2^ were treated with meniscal regularization or micro-drilling, respectively. Micro-drilling was performed with a low speed 1.2 mm K wire. At the end of the surgical procedure, aMFAT was injected in dry condition and no drainage was used in any of the cases included. The amount of the product injected was about 7 ml [18–20]. All patients started passive knee motion from first day post-operatively. Gradual weight bearing was allowed with crutches from third to fifth day post-operatively in all patients except those undergoing micro-drilling. In these cases, full weight bearing was allowed at 30 days after the surgical procedure.

### Study population

In all, 49 patients (50 knees) were enrolled for study assessment. No patients were excluded. The mean age of the selected cohort was 57.2 years (Standard Deviation (SD) 10.0 years, range: min. 39 – max. 76). Kellgren Lawrence radiographic classification was used to assess the severity of knee OA. Surgical records were reviewed for all cases and intraoperative chondral lesions were graded according to the Outerbridge classification [23]. Study population characteristics, preoperative diagnosis, knee OA classification, chondral lesion classification and locations were collected (Table 1). The mean radiographic anatomical lower limb alignment (aFTA), as measured on preoperative knee radiographs, was 183.5° (SD 4.2°).

**Table 1:** Population characteristics

Cases (knees)	Gender		Pre-operative diagnosis				Kellgren Lawrence Classification		Outerbridge Classification				Lesion Location						Surgical Procedure Performed		
	M	F	Post-traumatic OA	Rheumatoid Arthritis	Primitive OA	Post meniscectomy OA	III	IV	I	II	III	IV	Medial Compartmnet	Lateral Compartment	Patellofemoral Compartment	Medial + Lateral Compartment	Medial + Patellofemoral Compartment	Tricompartmental	Debridement	Selective meniscectomy	Microdrilling
49 (50)	24	25	2	2	42	4	2	48	0	4	17	29	20	5	6	3	7	9	24	18	8

### Patients reported outcome measures (PROMs)

All patients were evaluated pre- and post-operatively with the subjective International Knee Documentation Committee (IKDC) and the Knee Injury and Osteoarthritis Outcome Score (KOOS), which were the primary outcome measures [24, 25]. All patients were evaluated at one, three, six, 12 and 24 months post-operatively. PROMs were collected at one and 2 years post-operatively, as well as postoperative complications and cases of knee arthroplasty schedule. Patients were divided into three clusters based on their age and in two clusters based upon the complexity of arthroscopic procedures performed.

### Statistical analysis

Descriptive statistics were presented, including absolute numbers and percentages for categorical data and means and SD for continuous variables.

The correlation to determine the effect of the intraoperative Outerbridge classification (grade < 4 vs grade = 4), age (age ≤ 50 vs 50 < age < 60 vs age ≥ 60) and complexity of surgery (minor procedure - cartilage debridement and or meniscal regularization - vs major procedure - selective meniscectomy and/or condylar micro-drilling) on PROMs were carried out by using multivariable logistic regression models. The dependent variables were the postoperative outcome scores at one and 2 years, while the independent variables were the class of interest and the preoperative outcome score. Results were expressed as preoperative score-adjusted mean differences (MD) with 95% confidence intervals. Moreover, the number of patients that reached the Minimal Clinically Important Difference (MCID) was calculated. MCID was ten points for KOOS overall score and 16.7 for IKDC score [24, 25]. All tests were two-sided with a *p* value < 0.05 defining significance. Analyses were performed with R 3.4.3 software (The R Foundation for Statistical Computing, Wien).

## Results

A total of 50 knees were evaluated at baseline, at 1 year and 2 years post-operatively. Out of the 49 subjects enrolled, four were not evaluated at 1 year follow-up visit because they underwent knee arthroplasty. Three patients underwent total knee arthroplasty (TKA) and one unicompartmental knee arthroplasty (UKA). No subjects withdrew from study assessment, nor were lost at follow-up, hence a total of 46 knees were evaluated at the 2 years follow-up visit.

No major adverse reactions or complications were described neither at the study knee nor at the harvesting site, while five minor complications (one abdomen hematoma, four knee swelling) were recorded and recovered spontaneously without further treatment. After deduction of failures, cases grouped based upon age resulted as follows: age ≤ 50 years – 12 cases, 50 years < age < 60 years – 16 cases, and age ≥ 60 years – 18 cases. Arthroscopic complexity-based groups were: minor procedures – 21 cases, major procedures – 25 cases. Cases grouped based upon intraoperative Outerbridge classification of the most severe lesion were: grade II – four knees, grade III – 17 knees and grade IV – 25 cases. In all cases, IKCD and overall KOOS scores at one and 2 years of follow-up showed significant improvement compared to baseline values (Table 2). Except for KOOS Function in Sports and Recreation (FSR), decreasing at 2 years of follow-up (*p* < 0.01), no overall differences were reported between outcome scores at one and 2 years post-operatively. Globally, 42 (84%) knees reached the MCID for overall KOOS score at 1 year and 40 (80%) at 2 years, whereas the number of knees that reached the MCID for IKDC score was 37 (74%) at 1 year and 38 (76%) at 2 years. The mean outcome scores at baseline, one and 2 years post-operatively divided in groups based upon age, associated surgical procedures and chondral lesions are reported in the Appendix section. At 1 year after surgery patients younger than 50 years had higher scores in the KOOS subscales of FSR compared to patients aged between 50 and 60 (*p* < 0.01) and older than 60 (*p* < 0.05). Likewise, patients younger than 50 years had higher Quality of Life (QoL) scores compared to patients aged between 50 and 60 years (*p* < 0.05). At 2 years, patients with Outerbridge grade IV cartilage degeneration had worse scores in IKDC (*p* < 0.01), KOOS overall score and KOOS subscales Function in Activities of Daily Living (FD) (*p* < 0.05), FSR (*p* < 0.05) and QoL (*p* < 0.05) compared to patients with Outerbridge II and Outerbridge III (Table 3).

**Table 2:** Preoperative, one year and two years follow-up values for IKDC and KOOS overall and subcategories

	Pre-Operative	1 Year Follow-Up			2 Years Follow-Up		
	Mean ± SD (min-max)	Mean ± SD (min-max)	Improvement (MD) Vs Pre-Op	P value	Mean ± SD (min-max)	Improvement (MD) Vs Pre-Op	P value
IKDC	40.3 ± 10.3 (19.5–56.3)	66.6 ± 8.6 (37.9–95.4)	26.4	< 0.0001	66.8 ± 17.2 (19.5–89.7)	26.5	< 0.0001
KOOS Overall	48.3 ± 13.9 (23.2–73.1)	77.8 ± 17.1 (52.4–96.3)	29.5	< 0.0001	77.3 ± 23.8 (24.4–97.6)	29.0	< 0.0001
KOOS SS	53.6 ± 17.9 (7.1–85.7)	78.8 ± 11.9 (53.6–92.9)	25.3	< 0.0001	82.0 ± 22.5 (35.7–100.0)	28.4	< 0.0001
KOOS P	41.6 ± 15.0 (25.0–80.6)	77.9 ± 26.4 (5.0–94.4)	36.3	< 0.0001	77.8 ± 26.5 (5.0–97.2)	35.9	< 0.0001
KOOS FD	54.6 ± 13.5 (26.5–75.0)	81.8 ± 29.5 (60.3–98.5)	27.1	< 0.0001	82.1 ± 29.0 (25.0–100.0)	27.4	< 0.0001
KOOS FSR	31.4 ± 15.5 (0.0.55.0)	63.1 ± 25.3 (25.0–95.0)	31.8	< 0.0001	54.0 ± 28.4 (0.0–95.0)	22.7	< 0.0001
KOOS QoL	47.3 ± 14.2 (25.0–75.0)	69.7 ± 36.3 (37.5–100.0)	22.3	< 0.0001	68.6 ± 35.9 (6.3–100.0)	21.5	< 0.0001

**Table 3:** The difference in outcome values at one year and at two years follow-up

Time-point	1 year						2 years						
**Outcome**	**Variable**	**Comparison**	**MD**	**95%CI**		**p**	**Outcome**	**Variable**	**Comparison**	**MD**	**95%CI**		**p**
**IKDC**	**Age class**	B vs A	-4,06	−11,89	3,76	0,3161	**IKDC**	**Age class**	B vs A	−4,35	−16,81	8,11	0,4982
		C vs A	−5,05	−12,70	2,60	0,2047			C vs A	−6,27	−18,44	5,91	0,3202
	**Surgery Type**	2† vs 1*	−3,77	−10,18	2,64	0,2569		**Surgery Type**	2† vs 1*	6,96	−3,24	17,16	0,1900
	**Outerbridge Grade**	4 vs < 4	−2,25	−8,71	4,21	0,4994		**Outerbridge Grade**	4 vs < 4	−14,56	−24,84	−4,28	0,00889*‡*
**KOOS Overall**	**Age class**	B vs A	−6,93	−14,17	0,31	0,0691	**KOOS Overall**	**Age class**	B vs A	−8,85	−21,38	3,69	0,1758
		C vs A	−5,48	−12,29	1,32	0,1236			C vs A	−8,84	−20,64	2,95	0,1508
	**Surgery Type**	2† vs 1*	−1,93	− 7,69	3,84	0,5168		**Surgery Type**	2† vs 1*	5,55	−4,43	15,53	0,2831
	**Outerbridge Grade**	4 vs < 4	−0,47	−6,22	5,27	0,8723		**Outerbridge Grade**	4 vs < 4	−12,56	−22,50	−2,61	0,0185*‡*
**KOOS SS**	**Age class**	B vs A	−6,14	−13,67	1,38	0,1189	**KOOS SS**	**Age class**	B vs A	−10,23	−21,29	0,82	0,0784
		C vs A	−2,75	−9,97	4,47	0,4609			C vs A	−6,86	−17,46	3,75	0,2138
	**Surgery Type**	2† vs 1*	−1,56	−7,60	4,47	0,6148		**Surgery Type**	2† vs 1*	3,55	−5,31	12,42	0,4377
	**Outerbridge Grade**	4 vs < 4	0,72	− 5,30	6,73	0,8170		**Outerbridge Grade**	4 vs < 4	−9,09	−17,93	−0,26	0,0516
**KOOS P**	**Age class**	B vs A	−7,75	−19,18	3,69	0,1930	**KOOS P**	**Age class**	B vs A	−10,54	−26,95	5,87	0,2166
		C vs A	−3,58	−14,37	7,21	0,5197			C vs A	−6,68	−22,16	8,80	0,4034
	**Surgery Type**	2† vs 1*	−8,34	−17,55	0,88	0,0852		**Surgery Type**	2† vs 1*	−1,08	−14,31	12,14	0,8732
	**Outerbridge Grade**	4 vs < 4	5,03	−4,08	14,14	0,2866		**Outerbridge Grade**	4 vs < 4	−3,92	−16,99	9,16	0,5610
**KOOS FD**	**Age class**	B vs A	−6,30	−13,11	0,52	0,0792	**KOOS FD**	**Age class**	B vs A	−7,16	−20,01	5,69	0,2823
		C vs A	−4,64	−11,07	1,79	0,1664			C vs A	−9,66	−21,79	2,46	0,1274
	**Surgery Type**	2† vs 1*	−2,23	−7,68	3,21	0,4265		**Surgery Type**	2† vs 1*	3,66	−6,60	13,92	0,4890
	**Outerbridge Grade**	4 vs < 4	−1,70	−7,19	3,79	0,5473		**Outerbridge Grade**	4 vs < 4	−12,66	−23,01	−2,32	0,0221*‡*
**KOOS FSR**	**Age class**	B vs A	−17,97	−30,17	−5,76	0,0067*‡*	**KOOS FSR**	**Age class**	B vs A	−8,95	−27,32	9,42	0,3464
		C vs A	−13,00	−24,38	−1,62	0,0319*‡*			C vs A	−8,35	−25,49	8,78	0,3459
	**Surgery Type**	2† vs 1*	2,13	−7,53	11,79	0,6680		**Surgery Type**	2† vs 1*	14,79	0,25	29,33	0,0542
	**Outerbridge Grade**	4 vs < 4	−3,70	−13,32	5,91	0,4554		**Outerbridge Grade**	4 vs < 4	−17,74	−32,21	− 3,27	0,0219*‡*
**KOOS QoL**	**Age class**	B vs A	−10,68	−19,74	− 1,63	0,0269*‡*	**KOOS QoL**	**Age class**	B vs A	−15,00	−31,34	1,35	0,0811
		C vs A	−7,81	−16,53	0,91	0,0883			C vs A	−10,52	−26,26	5,22	0,1991
	**Surgery Type**	2† vs 1*	−2,08	−9,42	5,26	0,5823		**Surgery Type**	2† vs 1*	5,92	−7,33	19,17	0,3875
	**Outerbridge Grade**	4 vs < 4	−0,85	−8,20	6,49	0,8209		**Outerbridge Grade**	4 vs < 4	−14,86	−28,12	−1,61	0,0349*‡*

## Discussion

**Fig. 1:**
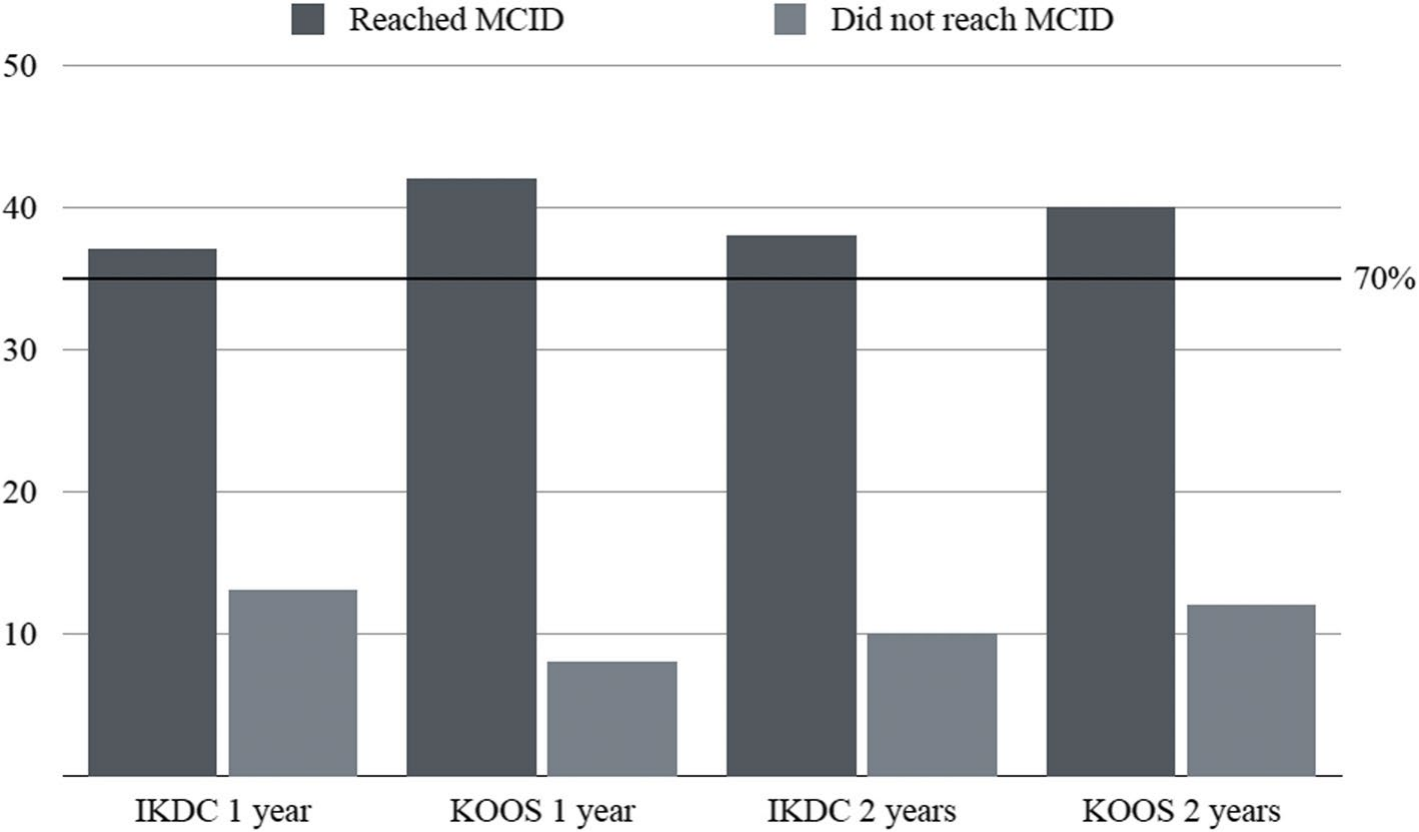
Percentage of patients reaching MCID for IKDC and KOOS at one- and two-years follow-up. Dark grey: patients who reached MCID; Light grey: patients who did not reached MCID; Horizontal line: 70% cut-off reached by both scores. MCID: Minimal Clinically Important Difference; IKDC: International Knee Documentation Committee; KOOS: Knee Injury and Osteoarthritis Outcome Score

In this retrospective, observational, single-center study the clinical performance of subjects with symptomatic Kellgren Lawrence III and IV knee OA undergoing aMFAT intra-articular knee injection in association with knee arthroscopy was assessed at one and 2 years post-operatively. The most important finding of the present study was that patients subjected to single injection of aMFAT and knee arthroscopy for symptomatic knee OA reported a substantial improvement in IKDC and KOOS scores in more than 70% of the sample both at one and 2 years post-operatively (*p* < 0.0001) (Fig. 1).

Patients without subchondral bone exposition had better results in IKDC, KOOS and 3 out of 5 KOOS subscales (FD, FSR and QoL) at 2 years follow-up. The findings reported by Borić et al., who showed increased glycosaminoglycans (GAG) content in cartilage after a single injection of aMFAT where at least a thin layer of cartilage was present [20], may explain the better outcomes in patients without subchondral bone expositions (Outerbridge < 4 vs =4).

The studied procedure confirmed to be safe, with no major associated complications. In accordance with literature, knee swelling occurred in four patients (8%), probably due to synovial membrane reaction to aMFAT [26, 27]. When swelling occurred, it always resolved spontaneously, thus knee arthrocentesis in the first 6 weeks from surgery should be avoided unless clinical signs of infection are present.

For what concerns failures, four patients (8% of all cases) required knee arthroplasty (one UKA and three TKAs) at 2 years follow-up. Similar results were reported by Borić et al. (9.4%) [20] and by Gobbi et al. (11.7%) [28], who performed a single aMFAT injection without arthroscopy.

Some papers advise against the benefit of knee arthroscopy in OA at mid to long term follow-up [29]. However, others showed better outcomes in patients treated with arthroscopy compared with physical therapy alone [30]. In addition, Kalunian et al. found beneficial effects on knee pain at 12 months of follow up when abundant irrigation prior arthroscopy procedure was performed [31]. The use of aMFAT in knee OA acts as a large-scale tool to supply damaged tissues with a regenerative environment [16]. For those reasons, we believe that washing away debris and recreating a “clean” environment in the knee prior to aMFAT injection might enhance its effect.

Boyd et al., analyzing a cohort of 68,090 patients, reported a 13.7% rate of TKA after knee arthroscopy alone at 2 years, with increased rates for patients older than 70 years (17.6%) [32].

Other authors have investigated the role of blood-derived products in delaying knee replacement. Sánchez et al. reported a delay for TKA of more than 1.5 years in 74.2% of the patients with multiple injections of PRP [33], while in the present study it was possible in 92% of the study cohort for at least 2 years.

Although with inferior results compared to patients with Outerbridge II and III chondral defects, the injection of aMFAT in patients with Outerbridge grade IV defects appeared to allow a temporary relief from symptoms. Given the satisfactory PROMs compared to baseline conditions in patients with Outerbridge grade IV lesion, the proposed technique could be useful in pain relief and improving symptoms and stiffness in end stage knee OA. For these reasons, bone-to-bone contact should be considered only a relative contraindication to this procedure [22].

Recently, Cattaneo et al. reported a decrease in pain and function limitation at 6 months and at 1 year after the described technique in association with arthroscopy [34].

Untreated knee OA has always been described as a progressive disease [35]. While TKA may be the gold-standard treatment option for end-stage knee OA, intra-articular injections have shown significant benefits in some patients, while none in others. A category of “responder” patients has been identified for both hyaluronic acid (HA) and PRP injections [36, 37]. Recently published meta-analyses comparing PRP, HA and steroids show a better overall effect of PRP compared to non-biological treatments [38, 39]. However, PRP seems to have inferior results compared to aMFAT combined with knee arthroscopy. Cole et al. reported a mean IKDC of 57.6 (SD 3.4) at 1 year after PRP injection [40], while the mean IKDC at 1 year reported in the present study was 66.6 (SD 8.6). Cole et al. also confirmed a significant effect of preoperative KL grade on IKDC, with higher KL grades having inferior IKDC scores. A double blind randomized self-controlled trial compared aMFAT to HA in patients with bilateral symptomatic KL grade II and III knee OA. Both treatments were effective against pain at first, but at 3 months HA lost efficacy and showed a significative worsening compared to baseline conditions. On the other hand, aMFAT showed longer effect, with persistent benefit over pain at 1 year after treatment [41]. These data are in accordance with the findings of the present study, showing improved KOOS Pain scores at one and 2 years of follow-up regardless of age, type of surgical procedure and chondral lesion.

Several limitations should be discussed before drawing the conclusions from the present study. The most important study limitation is the absence of a control group due to the retrospective nature of the study. Moreover, the associated surgical procedure is the main confounding factor for the clinical benefit assessment. However, the reported outcomes are still interesting because arthroscopy by itself does not produce any statistically relevant improvement than placebo or physical therapies in moderate-severe knee OA at mid-term [29, 30]. Also, a relevant limitation is represented by the absence of sample size calculation and randomization. Further study with larger population and longer follow-ups is needed to confirm the promising results.

## Conclusion

The injection of aMFAT associated with arthroscopy for the treatment of symptomatic KL III and IV knee OA demonstrated to be a safe and effective technique. Improvements at 2 years post-operatively were reported by all the categories of patients, with no correlation with age and complexity of surgical procedure. Higher clinical benefits were reported by those patients having residual cartilage layers (Outerbridge grade < 4).

## Supplementary Information

**Additional file 1.** Appendix: The mean outcome scores pre-operatively, at 1 year and 2 years follow-up. Legend: All values are expressed as absolute values. Mean values ± SD and min-max are expressed for each age group (≤50, 50–60, ≥60 years), complexity of surgery (type = 1*: debridement or meniscal regularization; type = 2†: selective meniscectomy and/or micro-drilling) and Outerbridge classification (< 4: Outerbridge grade II and III; = 4: Outerbridge grade IV). All values are expressed as absolute values. IKDC: International Knee Documentation Committee, KOOS: Knee Injury and Osteoarthritis Outcome Score, SS: Symptoms and Stiffness, P: Pain, FD: Function in activities of Daily living, FSR: Function in Sports and Recreation, QoL: Quality of Life, SD: standard deviation, min-max: minimum-maximum.

## Data Availability

The datasets generated and/or analyzed during the current study are not publicly available due to privacy reasons but are available from the corresponding author upon reasonable request.

## Abbreviations

aFTA: Anatomical Femoro-Tibial Angle
aMFAT: Autologous Micro-Fragmented Adipose Tissue
APS: Autologous Protein Solution
F: Female
FD: Function in Activities of Daily Living
FSR: Function in Sports and Recreation
GAG: Glycosaminoglycans
HA: Hyaluronic Acid
IKDC: Subjective International Knee Documentation Committee
KL: Kellgren Lawrence
KOOS: Knee Injury and Osteoarthritis Outcome Score
M: Male
MCID: Minimal Clinically Important Difference
MD: Mean Difference
min-max: Minimum-maximum
MSCs: Mesenchymal Stem Cells
OA: Osteoarthritis
P: Pain
PROMs: Patients Reported Outcome Measures
PRP: Platelet Rich Plasma
QoL: Quality of Life
SD: Standard Deviation
SS: Symptoms and Stiffness
TKA: Total knee arthroplasty
UKA: Unicompartmental Knee Arthroplasty
