# Supplementary material for: Autologous micro-fragmented adipose tissue associated with arthroscopy in moderate–severe knee osteoarthritis: outcome at two year follow-up
Source: BMC Musculoskelet Disord. 2022 Nov 8;23:963. doi: 10.1186/s12891-022-05921-6 (PMC9641752; doi:10.1186/s12891-022-05921-6)
Supplement: Supplementary file 1 — Additional file 1. Appendix: The mean outcome scores pre-operatively, at 1 year and 2 years follow-up. Legend: All values are expressed as absolute values. Mean values ± SD and min-max are expressed for each age group (≤50, 50–60, ≥60 years), complexity of surgery (type = 1*: debridement or meniscal regularization; type = 2†: selective meniscectomy and/or micro-drilling) and Outerbridge classification (< 4: Outerbridge grade II and III; = 4: Outerbridge grade IV). All values are expressed as absolute values. IKDC: International Knee Documentation Committee, KOOS: Knee Injury and Osteoarthritis Outcome Score, SS: Symptoms and Stiffness, P: Pain, FD: Function in activities of Daily living, FSR: Function in Sports and Recreation, QoL: Quality of Life, SD: standard deviation, min-max: minimum-maximum. [file 12891_2022_5921_MOESM1_ESM.doc]

Appendix: The mean outcome scores pre-operatively, at one year and two years follow-up

|  | | IKDC | KOOS Overall | KOOS SS | KOOS P | KOOS FD | KOOS FSR | KOOS QoL |
| --- | --- | --- | --- | --- | --- | --- | --- | --- |
|  |  | Mean ± SD  (min. - max.) | Mean ± SD  (min. - max.) | Mean ± SD  (min. - max.) | Mean ± SD  (min. - max.) | Mean ± SD  (min. - max.) | Mean ± SD  (min. - max.) | Mean ± SD  (min. - max.) |
| Time-point | | Pre operative | | | | | | |
| Age | < 50 years | 41.2 ± 9.1 (24.1 - 50.6) | 45.9 ± 12.8 (28.0 - 64.9) | 52.5 ± 17.4 (32.1 - 82.1) | 38.6 ± 13.8 (25.0 - 63.9) | 52.4 ±13.3 (33.8 - 73.5) | 28.0 ± 14.6 (0.0 - 45.0) | 45.6 ± 14.1 (25 - 68.8) |
|  | 50 < years < 60 | 43.0 ± 10.3 (20.7 - 55.2) | 54.2 ± 14.5 (23.2 - 73.1) | 61.2 ± 16.3 (28.6 - 85.7) | 46.8 ± 15.4 (25.0 - 72.2) | 59.8 ± 14.4 (26.5 - 73.5) | 38.2 ± 15.3 (0.0 - 50.0) | 51.8 ± 16.5 (25.0 - 75.0) |
|  | years > 60 | 37.3 ± 10.9 (19.5 - 50.6) | 44.7 ± 13.1 (23.2 - 70.6) | 47.5 ± 18.0 (7.1-75.0) | 38.9 ± 14.9 (25.0 - 80.6) | 51.6 ± 12.3 (32.4 - 75.0) | 27.5 ± 15.1 (0.0 -50.0) | 44.5 ± 11.8 (25.0 - 68.8) |
| Complexity of surgery | Type 1* | 40.9 ± 10.9 (23.0 - 56.3) | 49.4 ± 15.3 (23.2 - 73.1) | 54.1 ± 20.2 (7.1 -85.7) | 43.7 ± 16.8 (25.0 - 80.6) | 55.2 ± 14.7 (32.4 - 75.0) | 32.8 ± 14.2 (0.0 - 55.0) | 46.9 ± 15.3 (25.0 - 75.0) |
|  | Type 2† | 39.7 ± 10.1 (19.5 - 52.9) | 47.5 ± 12.9 (23.2 - 70.8) | 53.1 ± 16.2 (25.0 - 82.1) | 39.9 ± 13.4 (25.0 - 72.2) | 54.2 ± 12.9 (26.5 - 73.5) | 30.2 ± 16.7 (25.0 - 75.0) | 47.7 ± 13.6 (25.0 - 75.0) |
| Outerbridge classification | < 4 | 38.7 ± 10.9 (19.5 - 55.2) | 47.6 ± 15.5 (23.2 - 73.1) | 54.0 ± 21.3 (7.1 - 85.7) | 40.9 ± 14.8 (25.0 - 63.9) | 52.7 ± 15.0 (26.5 - 73.5) | 30.6 ± 14.5 (0.0 - 55-0) | 49.3 ± 15.9 (25.0 -75.0) |
|  | 4 | 41.4 ± 10.0 (23.0 - 56.3) | 48.8 ± 13.0 (28.0 - 70.8) | 53.2 ± 15.4 (25.0 - 82.1) | 42.1 ± 15.4 (25.0 - 80.6) | 56.1 ± 12.5 (35.3 - 75.0) | 32.0 ± 16.5 (0.0 - 55.0) | 45.9 ± 13.0 (25.0 - 75.0) |
| Time-point | | 1 year follow-up | | | | | | |
| Age | < 50 years | 71.0 ± 15.8 (40.2 - 95.4) | 82.0 ± 10.4 (60.1 - 96.3) | 82.1 ± 9.7 (64.3 - 92.9) | 82.5 ± 10.3 (58.3 - 94.4) | 85.5 ± 9.5 (64.7 - 98.5) | 72.0 ± 16.9 (40.0 - 95.0) | 76.3 ± 14.4 (56.9 - 100.0) |
|  | 50 < years < 60 | 67.5 ± 5.3 (56.3 - 73.6) | 77.2 ± 7.0 (63.1 - 86.3) | 77.3 ± 7.6 (60.7 - 89.3) | 75.4 ± 20.8 (5.0 - 88.9) | 81.0 ± 6.4 (66.2 - 89.7) | 61.4 ± 13.1 (40.0 - 85.0) | 67.4 ± 6.1 (56.3 - 75.0) |
|  | years > 60 | 63.2 ± 10.4 (37.9 - 79.3) | 75.7 ± 10.0 (52.4 - 88.7) | 78.1 ± 10.1 (53.8 - 89.3) | 77.3 ± 9.0 (88.3 - 98.9) | 80.1 ± 9.5 (60.3 - 92.7) | 59.1 ± 19.2 (25.0 - 85.0) | 67.6 ± 13.1 (37.5 - 81.3) |
| Complexity of surgery | Type 1* | 69.9 ± 10.2 (52.9 - 95.4) | 79.7 ± 8.6 (61.3 - 96.3) | 80.0 ± 8.2 (64.3 - 92.9) | 82.6 ± 6.6 (69.4 - 94.4) | 83.9 ± 7.8 (64.7 - 98.5) | 64.7 ± 16.6 (35.0 - 95-0) | 71.5 ± 12.2 (62.5 - 100.0) |
|  | Type 2† | 64.0 ± 11.0 (37.9 - 79.3) | 76.2 ± 9.7 (52.4 - 88.7) | 77.9 ± 10.0 (53.6 - 89.3) | 74.1 ± 17.8 (5.0 - 89.9) | 80.1 ± 9.1 (60.3 -92.7) | 61.8 ± 17.8 (25.0 - 85.0) | 68.2 ± 11.7 (37.5 - 75.0) |
| Outerbridge classification | < 4 | 67.9 ± 12.3 (37.9 - 95.4) | 78.1 ± 10.4 (52.4 - 96.3) | 78.6 ± 10.6 (53.6 - 92.9) | 76.1 ± 20.1 (5.0 - 94.4) | 82.5 ± 9.5 (60.3 - 98.5) | 63.8 ± 18.8 (25.0 - 95.0) | 71.0 ± 14.0 (37.5 - 100.0) |
|  | 4 | 65.7 ± 9.9 (40.2 - 79.3) | 77.6 ± 8.6 (60.1 - 88.7) | 79.0 ± 8.2 (64.3 - 89.3) | 79.2 ± 8.4 (58.3 -88.9) | 81.2 ± 8.1 (64.7 - 92.7) | 62.6 ± 16.1 (35.0 - 85.0) | 68.8 ± 10.3 (50.0 - 81.3) |
| Time-point | | 2 years follow-up | | | | | | |
| Age | < 50 years | 70.0 ± 12.8 (36.8 - 81.6) | 81.4 ± 8.1 (59.5 - 88.1) | 87.1 ± 10.0 (64.3 -100.0) | 82.8 ± 7.2 (63.9 - 88.9) | 86.3 ± 8.7 (67.7 - 95.6) | 55.5 ± 15.4 (30.0 - 75.0) | 75.6 ± 14.3 (43.8 - 93.8) |
|  | 50 < years < 60 | 69.6 ± 10.7 (47.1 - 88.5) | 79.5 ± 10.9 (54.3 - 95.2) | 81.6 ± 10.1 (60.7 - 100.0) | 76.4 ± 24.1 (5.0 - 97.2) | 85.4 ± 9.9 (66.2 -97.1) | 57.9 ± 19.8 (5.0 - 85.0) | 67.4 ± 16.1 (25.0 - 93.8) |
|  | years > 60 | 62.2 ± 22.8 (19.5 - 89.7) | 72.9 ± 23.5 (24.4 - 97.6) | 79.0 ± 19.6 (35.7 -100.0) | 75.9 ± 21.0 (27.8 - 97.2) | 76.7 ± 24.2 (25.0 - 100.0) | 49.7 ± 31.0 (0.0 -95.0) | 65.3 ± 30.4 (6.3 - 100.0) |
| Complexity of surgery | Type 1* | 66.6 ± 14.6 (29.9 - 88.5) | 77.9 ± 12.5 (45.8 - 95.2) | 82.5 ± 10.9 (50.0 - 100.0) | 80.7 ± 13.2 (27.8 - 97.2) | 83.6 ± 12.7 (51.5 - 95.6) | 50.8 ± 18.5 (10.0-75.0) | 68.8 ± 20.9 (37.5 - 100.0) |
|  | Type 2† | 66.9 ± 19.2 (19.5 - 89.7) | 76.8 ± 19.8 (24.4- 97.6) | 81.5 ± 17.4 (37.5 - 100.0) | 75.4 ± 23.6 (5.0 - 97.2) | 80.9 ± 20.4 (25.0 - 100.0) | 56.6 ± 27.6 (0.0 - 95.0) | 68.5 ± 24.3 (6.3 - 100.0) |
| Outerbridge classification | < 4 | 72.7 ± 9.8 (56.3 - 88.5) | 82.9 ± 9.0 (63.1 - 97.6) | 86.3 ± 9.8 (60.7 - 100.0) | 79.5 ± 21.4 (5.0 - 97.2) | 87.5 ± 8.6 (66.2 -100.0) | 60.6 ± 17.4 (30.0 - 90.0) | 76.9 ± 15.2 (50.0 - 100.0) |
|  | 4 | 62.4 ± 19.9 (19.5 - 89.7) | 73.2 ± 19.9 (24.4 - 94.0) | 78.7 ± 16.9 (35.7 - 100.0) | 76.4 ± 18.5 (27.8 - 97.2) | 78.1 ± 20.8 (25.0 - 97.1) | 49.1 ± 27.0 (0.0 - 95.0) | 62.5 ± 25.3 (6.3 - 93.8) |
